# Perioperative and long-term outcomes of liver resection for hepatitis B virus-related hepatocellular carcinoma without versus with hepatic inflow occlusion: study protocol for a prospective randomized controlled trial

**DOI:** 10.1186/s13063-016-1621-9

**Published:** 2016-10-11

**Authors:** Yinzhe Xu, Jiye Chen, Hongguang Wang, Hui Zheng, Dan Feng, Aiqun Zhang, Jianjun Leng, Weidong Duan, Zhanyu Yang, Mingyi Chen, Xianjie Shi, Shouwang Cai, Wenbin Ji, Kai Jiang, Wenzhi Zhang, Yongliang Chen, Wanqing Gu, Jiahong Dong, Shichun Lu

**Affiliations:** 1 Department of Hepatobiliary Surgery, Chinese People’s Liberation Army General Hospital, 28 Fuxing Road, Haidian, Beijing 100853 China; 2 Division of Transplantation, Department of Surgery, Massachusetts General Hospital, Harvard Medical School, 55 Fruit Street, Boston, MA 02114 USA; 3 Biostatistics Center, Massachusetts General Hospital, Harvard Medical School, 50 Staniford Street, Boston, MA 02114 USA; 4 Division of Standard Operational Management, Institute of Hospital Management, Chinese PLA General Hospital, 28 Fuxing Road, Haidian, Beijing 100853 China

**Keywords:** Hepatocellular carcinoma, Liver resection, Vascular occlusion, Randomized controlled trial, Ischemia-reperfusion injury, Inflammatory response, Survival

## Abstract

**Background:**

The high prevalence of hepatitis B virus (HBV) imposes a huge burden of hepatocellular carcinoma (HCC) in Asia. Surgical resection remains an important therapeutic strategy for HCC. Hepatic inflow occlusion, known as the *Pringle maneuver*, is the most commonly used method of reducing blood loss during liver parenchymal transection. A major issue with this maneuver is ischemia-reperfusion injury to the remnant liver, and the hemodynamic disturbance it induces in the tumor-bearing liver raises an oncological concern. Given the technical advances in living donor liver transplantation, vascular occlusion in liver resection can be avoided in experienced hands. The aim of this study is to compare the perioperative and long-term outcomes of liver resection for HBV-related HCC without versus with hepatic inflow occlusion.

**Methods/design:**

This study will include eligible patients with HBV-related HCC elected for liver resection. Fifty-seven patients will be enrolled in each randomization arm to detect a 20 % difference in the serum level of total bilirubin on postoperative day 5 (80 % power and α = 0.05). The secondary endpoints include procedural parameters, perioperative liver function and inflammatory response, postoperative morbidity and mortality, and long-term outcomes. Patients will be followed for up to 5 years. Data will be statistically analyzed on an intention-to-treat basis.

**Discussion:**

This prospective randomized controlled trial is designed to compare the perioperative and long-term outcomes of liver resection for HBV-related HCC without versus with vascular occlusion. The clinical implications of these outcomes may change current surgical practice and fill the oncological gaps therein.

**Trial registration:**

Clinicaltrials.gov identifier NCT02563158. Registered on 28 September 2015.

**Electronic supplementary material:**

The online version of this article (doi:10.1186/s13063-016-1621-9) contains supplementary material, which is available to authorized users.

## Background

Hepatocellular carcinoma (HCC) is the fifth most common neoplasm worldwide and the third leading cause of cancer mortality [1]. In China, HCC is the second most common cause of cancer-related death [2], and up to 80 % of HCC cases are attributable to hepatitis B virus (HBV) infection [3, 4].

Surgical resection remains an important therapeutic strategy for HCC [5]. Intraoperative blood loss and requirement for transfusion have been shown to correlate well with perioperative morbidity and mortality [6]. Hepatic inflow occlusion, also known as the *Pringle maneuver*, is traditionally practiced to reduce blood loss during liver parenchymal transection [7]. However, this maneuver induces significant ischemia-reperfusion (IR) injury to the remnant liver, especially in patients with underlying liver disease [8]. In China, hepatitis B virus surface antigen is detected in 70–79 % patients with cirrhosis of the liver [9, 10]. It is widely accepted that the cirrhotic liver is particularly sensitive to IR injury [11, 12]. Furthermore, intermittent application of the Pringle maneuver, a recommended practice, could induce hemodynamic disturbance to the tumor-bearing liver, which raises an oncological concern [13, 14].

In living donor liver transplant (LDLT), liver procurement is performed without hepatic vascular occlusion to reduce the graft warm ischemia time and the IR injury to the donor remnant liver [15, 16]. In recent years, liver resections, even those done for cirrhotic livers, have been performed without vascular occlusion in some centers with expertise [17, 18]. We therefore hypothesize that liver resection for HBV-related HCC (the most common etiology in our region) could be safely performed without vascular occlusion. The present prospective randomized controlled trial (RCT) is designed to compare the perioperative and long-term outcomes of liver resections without and with hepatic inflow occlusion.

## Methods/design

### Trial population

From January 2016 to November 2017, all patients with HBV-related HCC scheduled for elective partial hepatectomy in the Department of Hepatobiliary Surgery, Chinese People’s Liberation Army General Hospital (PLAGH), will be screened for eligibility for this trial.

#### Inclusion criteria

Patients who meet the following criteria will be included in the study:

1. Aged between 18 and 65 years
2. Elective liver resection for HBV-related HCC with Barcelona-Clínic Liver Cancer stage 0 or A [19]
3. Child-Pugh class A with or without cirrhosis, or reversed to class A from class B after conventional therapy
4. Tumors located in either the left or right liver lobe
5. Resection extent is a hemihepatectomy or less
6. Informed consent provided

#### Exclusion criteria

Patients who meet any of the following criteria will be excluded from the study:

1. Having comorbidity that contraindicates surgery
2. Undergoing intervention as part of other ongoing trials with interference with the present study
3. Having undergone nonsurgical interventions that included portal vein ligation/embolization, radiofrequency ablation, and transarterial chemoembolization
4. Scheduled for laparoscopic hepatectomy
5. Requiring concomitant procedures, such as digestive, vascular, or biliary reconstruction
6. Lack of compliance with treatment or future follow-up

### Study design

#### Randomization and blinding

Randomization will be performed to achieve comparability in terms of known and unknown confounding variables. The patient allocation will be based on computerized random number generation. All patients eligible for the study will be allocated an opaque envelope marked only with the patient-specific random number. The decision regarding the procedure performed will be sealed inside. The envelope will be opened right before the preoperative instruction for surgery is given to the patient, and then the patient will be automatically assigned to group A (with hepatic inflow occlusion) or group B (without hepatic inflow occlusion). Owing to the nature of surgery and the patient’s right of informed consent, neither the surgeons nor the patients can be blinded to the procedural details. Only the trial coordinators will be blinded to the information regarding patient allocation during data processing and analysis.

#### Withdrawal

Participants can withdraw at any time during the trial at their own or their legal representative’s request. A participant may also be withdrawn if, on the basis of the investigators’ judgment, continuation of the trial may be detrimental to the participant’s health. Reasons for all withdrawals will be recorded in patients’ medical files and their case report forms (CRFs). All data will be analyzed according to the intention-to-treat principle [20].

#### Ethics, study registration, and consent

The study protocol was approved by the PLAGH Medical Ethics Committee (S2015-081-01) and is registered in the ClinicalTrials.gov protocol registration system (NCT02563158). The study procedure, benefits, risks, and data management will be introduced in detail to all eligible participants before they are asked to provide written informed consent.

#### Data management and quality assurance

Two independent trial coordinators (YX and AZ), who will have no contact with any patient, will double-enter all required data from the CRF and medical records of patients. The CRF will be completed on the day of treatment and at the follow-up visits. Reasons for missing data will be recorded. The principal investigator and the responsible monitor will routinely check the completeness and plausibility of the CRF. The participants will be enrolled strictly according to the inclusion criteria. The surgeons, statisticians, and trial coordinators will be well-trained to avoid the possible risks of bias throughout this unmasked trial, such as unfair evaluation, selection, performance, and attrition. The attrition of the trial, including patient dropout and/or withdrawal and missing data, will be prevented and treated according to published recommendations [21]. The final report will follow the Consolidated Standards of Reporting Trials (CONSORT) 2010 guidelines as well as the extension to nonpharmacological interventions [22] (Additional files 1 and 2).

### Surgical interventions

#### Preoperative evaluation

Preoperative management will include imaging studies and laboratory serum tests (see Endpoints section below). Other virological and oncological tests to be performed include HBV antigens, antibodies, and DNA; hepatitis C virus antibody; serum alpha-fetoprotein (AFP); carcinoembryonic antigen; and carbohydrate antigen 19–9. The Child-Pugh grades for liver function will be determined on the basis of serum parameters and the absence or presence of ascites and hepatoencephalopathy [23]. The diagnosis of HCC will be based on the identification of typical radiological hallmarks of HCC (hypervascular in the arterial phase with washout in the portal venous or delayed phase) by four-phase multidetector computed tomography (CT) or dynamic contrast-enhanced magnetic resonance imaging (MRI) [5]. According to the practice recommended by the Ministry of Health of the People’s Republic of China [24], a more conservative approach with two imaging techniques or one technique plus AFP ≥400 ng/ml will be recommended in some suboptimal settings, such as when the nodule is <2 cm in diameter. Advanced imaging such as positron emission tomography will be performed in cases of suspicion of extrahepatic metastases.

#### Surgical procedures

The same group of surgeons with experience of over 3000 hepatectomies and 200 LDLTs will perform all the procedures. The abdominal cavity will be opened through a J-shaped subcostal incision and carefully searched for peritoneal seeding and extrahepatic metastases. After the liver is mobilized, an intraoperative ultrasound will be routinely performed to confirm preoperative findings and double-check the tumor and vascular anatomy.

#### Group A: liver resection with hepatic inflow occlusion

Liver resection will be carried out using the Pringle maneuver to occlude vascular inflow in cycles of 15 minutes of clamping plus 5 minutes of unclamping of the hepatoduodenal ligament. An elastic tourniquet will be used to encircle and tighten the hepatoduodenal ligament to occlude the hepatic blood inflow. A clamp will be used to fix the tourniquet. During parenchymal transection, a hybrid application of electronic cautery, cavitron ultrasound surgical aspirator, titanium clips, and silk ligation will be practiced in an institutional permutation. To reduce bleeding from the hepatic venous system, <5-mmHg central venous pressure will be maintained with the patient in a feet-down tilt position and with volume restriction [25]. After parenchymal transection, an argon beam coagulator will be used for hemostasis of the transection surface. Double-drains to the liver bed will be routinely placed.

#### Group B: liver resection without hepatic inflow occlusion

The procedural techniques used in group B will be same as those in group A, but without vascular occlusion. For the sake of comparability between groups and in cases of problematic bleeding, the hepatoduodenal ligament will still be encircled with an elastic tourniquet. Unplanned conversion from the nonocclusion group to the occlusion group and its cause will be recorded in the patient’s CRF and medical record.

#### Postoperative management

All patients will be transferred to the intensive care unit (ICU) of the PLAGH Department of Hepatobiliary Surgery for early postoperative monitoring. Any subsequent need for an ICU stay will depend on patient-specific status. Postoperative serum parameters will be monitored on a serial basis (see Endpoints section below).

#### Follow-up

Patient follow-up will start upon discharge from the hospital. All patients are required to visit their surgical team in person 3 and 6 months as well as 1, 2, 3, and 5 years after surgery or whenever necessary. Routine checkups, including abdominal ultrasound, CT and/or MRI, and serum biochemistry and AFP will be performed at each visit. Checkup data from qualified local hospitals will also be acceptable.

### Endpoints

#### Primary endpoint

The primary endpoint will be the serum level of total bilirubin (TBil) on postoperative day (POD) 5, which determines posthepatectomy liver failure (PHLF) in accordance with the guidelines published by the International Study Group of Liver Surgery (ISGLS) [26].

#### Secondary endpoints

The following will be the secondary endpoints:

1. Procedural parameters, including intraoperative blood loss, requirement for and amount of blood transfusion, liver transection time, and operative time
2. Perioperative liver function and inflammatory response, including serum levels of alanine aminotransferase (ALT); aspartate aminotransferase (AST); albumin (ALB); prothrombin time (PT); international normalized ratio (INR); tumor necrosis factor-α (TNF-α); interleukin (IL)-1α, IL-2, IL-6, IL-8, and IL-10; procalcitonin; and C-reactive protein (CRP) at serial time points (preoperative day and PODs 1, 3, 5, and 7)
3. Postoperative course, including complications defined by Clavien-Dindo classification [27], in-hospital and 90-day mortality, duration of ICU and hospital stays, and total in-hospital medical expenditure
4. Long-term outcomes, including 1-, 3-, and 5-year tumor recurrence rate; overall survival; and disease (tumor)-free survival (Table 1)

**Table 1:** Definition of endpoints

Endpoints	Definition
Primary	
Postoperative liver function	Serum TBil on POD 5 [26]
Secondary	
Procedural parameters	
Intraoperative blood loss	Total blood loss from skin incision to closure, including the amount of blood in the suction containers and the weight of absorptive materials after subtracting the rinse fluid and ascites
Requirement of blood transfusion	Indication: massive hemorrhage (>1500 ml) or hemoglobin level <7 g/dl; amount of transfusion
Liver transection time	Time from parenchymal dissection to removal of liver specimen (minutes)
Operative time	Time from skin incision to closure (in minutes)
Perioperative serum parameters	
Liver function	Serum ALT, AST, ALB, PT, and INR preoperatively and on PODs 1, 3, 5, and 7
Inflammatory response	Serum TNF-α, IL-1α, IL-2, IL-6, IL-8, IL-10, PCT, and CRP preoperatively and on PODs 1, 3, 5, and 7
Postoperative course	
Complications	Defined by Clavien-Dindo classification (I–IV) [27]
PHLF	Increased INR (or need of clotting factors to maintain normal INR) and hyperbilirubinemia on or after POD 5; if INR or serum bilirubin concentration is increased preoperatively, PHLF is defined by increasing INR and bilirubin concentration on or after POD 5 (biliary obstruction should be ruled out); graded according to ISGLS [26] as follows: A: PHLF requiring no or little change in patient’s clinical management B: PHLF resulting in deviation from regular clinical management but manageable without invasive treatment C: PHLF requiring invasive treatment
Bile leakage	Increased bilirubin concentration (at least three times greater than the serum level measured at the same time) in abdominal drain or intraabdominal fluid on or after POD 3, or as need for radiological intervention (e.g., interventional drainage) because of biliary collections or relaparotomy resulting from bile peritonitis; graded according to ISGLS [39] as follows: A: Bile leakage requiring no or little change in patient’s clinical management B: Bile leakage requiring change in patient’s clinical management (e.g., additional diagnostic or interventional procedures) but manageable without relaparotomy, or a grade A bile leakage lasting >1 week C: Bile leakage requiring relaparotomy
Posthepatectomy hemorrhage (PHH)	Evidence of intraabdominal bleeding such as frank blood loss via the abdominal drains (e.g., hemoglobin level in drain fluid >3 g/dl) or detection of intraabdominal hematoma or active hemorrhage by abdominal imaging (ultrasound, CT, angiography); graded according to ISGLS [40] as follows: A: PHH requiring transfusion of ≤2 U of PRBCs B: PHH requiring transfusion of >2 U of PRBCs but manageable without invasive intervention C: PHH requiring radiological interventional treatment (e.g., embolization) or relaparotomy
Intraperitoneal effusion/abscess	Any imaging-detected intraperitoneal fluid collection and/or elevation of infectious parameters (CRP >2 mg/dl and/or leukocytes >100,000/ml), positive physical signs, and bacteriology of abdominal drainage
Pulmonary infection	Elevation of infectious parameters (CRP >2 mg/dl and/or leukocytes >100,000/ml) and/or evidence of pulmonary infiltration on chest x-ray requiring antibiotic therapy
Postoperative ICU/hospital stay	Time from day of operation through discharge from ICU and/or hospital (days)
Total in-hospital expenditure	Costs from admission to discharge (¥/$)
Mortality	In-hospital death and 90-day death.
Long-term outcomes	
Survival	1-, 3-, and 5-year overall and disease (tumor)-free survival
Tumor recurrence	Identification of the typical hallmarks of recurrent HCC foci by dynamic imaging (CT/MRI) plus AFP >400 ng/ml in suboptimal settings (e.g., foci <2 cm) [24]

*Abbreviations: AFP* Alpha-fetoprotein, *ALB* Albumin, *ALT* Alanine aminotransferase, *AST* Aspartate aminotransferase, *CRP* C-reactive protein, *CT* Computed tomography, *HCC* Hepatocellular carcinoma, *ICU* Intensive care unit, *IL* Interleukin, *INR* International normalized ratio, *ISGLS* International Study Group of Liver Surgery, *MRI* Magnetic resonance imaging, *PCT* Procalcitonin, *PHH* Posthepatectomy hemorrhage, *PHLF* Posthepatectomy liver failure, *POD* Postoperative day, *PRBCs* Packed red blood cells, *PT* Prothrombin time, *TBil* Total bilirubin, *TNF-α* Tumor necrosis factor-α

### Sample size calculation

**Fig. 1:**
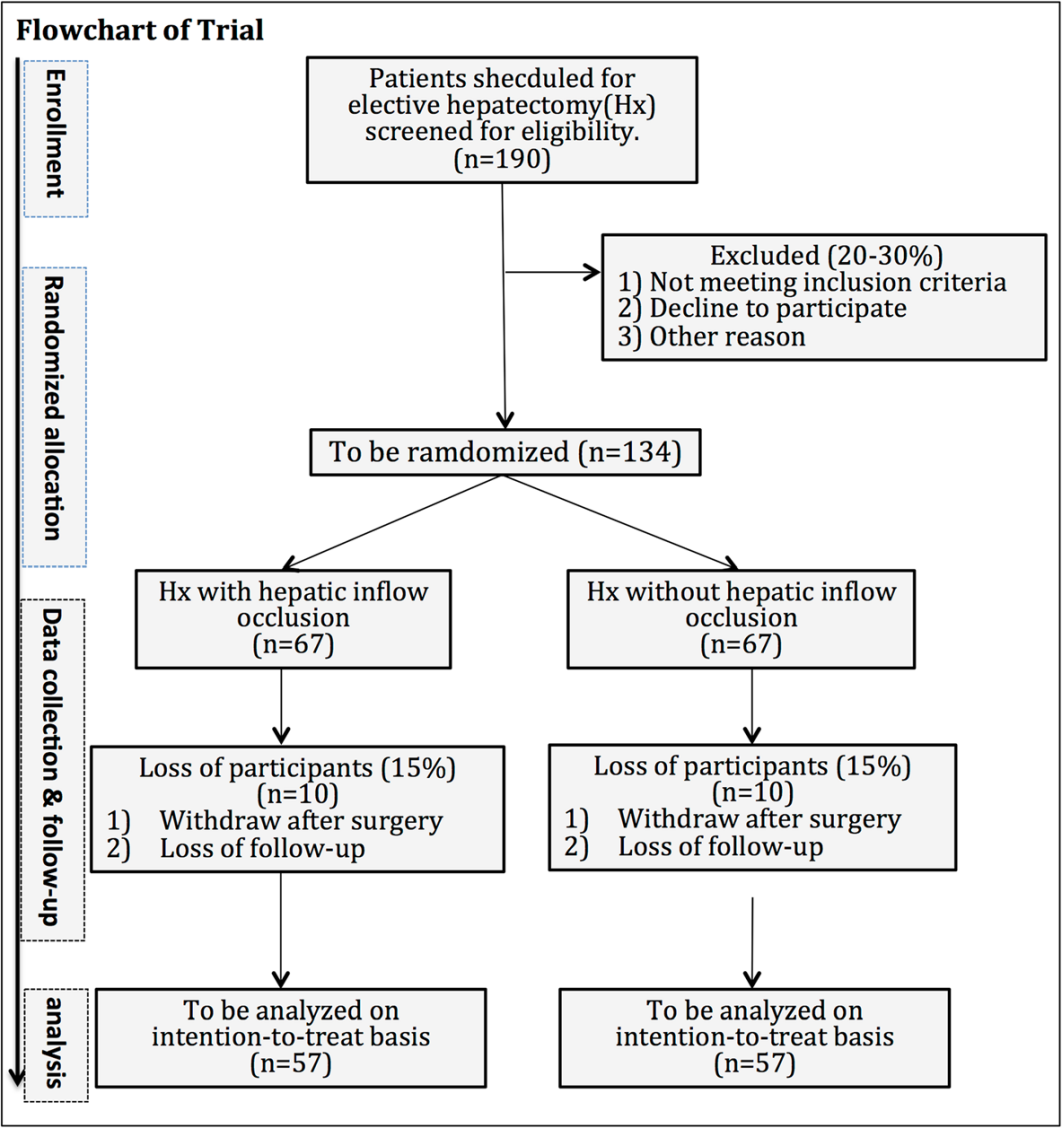
Flowchart of the trial

Our retrospective data for 17 patients with HBV-related HCC undergoing liver resection with hepatic inflow occlusion (Pringle maneuver) and 22 patients without inflow occlusion showed that the mean ± SD values of serum TBil on POD 5 were 19.2 ± 6.9 μmol/L in the occlusion group and 15.0 ± 5.3 μmol/L in the nonocclusion group (J. C., unpublished data). The sample size for the prospective trial is estimated on the basis of an expectation of a 20 % reduction in serum TBil on POD 5 [26, 28, 29]. On the basis of a two-samples *t* test with a statistical difference of 0.05 and a power of 1 − β = 0.90, a sample size of 57 patients in each randomization arm will be required. Assuming an estimated withdrawal, protocol violation, and loss to follow-up rate of 15 % during the trial, a total number of 134 patients need to be enrolled. The time frame of patient enrollment will be approximately 24 months (Fig. 1).

### Statistical analysis

The two-sided null hypothesis for the primary endpoint states that both interventions (liver resection with versus without hepatic inflow occlusion) lead to similar serum levels of TBil on POD 5. The alternative hypothesis states that one intervention performs better with lower TBil on POD 5 than the other. This null hypothesis will be tested by an analysis of covariance that adjusts for the baseline measures (e.g., presence of cirrhosis, preoperative TBil, and remnant liver volume estimated using CT). We will adjust for the differences in background characteristics by including them in a multivariable regression model. All background characteristics and surgical outcome measures will be presented as mean ± SD or median with range for the continuous variables, and as frequency or percentage for the categorical variables. A two-sided Student’s *t* test will be used to compare continuous parametric variables such as the serum TBil on POD 5, and a Mann–Whitney *U* test will be used to compare discrete or nonparametric variables, as appropriate. Categorical variables will be compared using the chi-square test or Fisher’s exact test. The homogeneity of patients in the two treatment arms will be described by comparing the demographic and baseline values. The serum parameters at serial time points will be analyzed using repeated-measures analysis of variance. The Kaplan-Meier method with log-rank test will be used for survival analysis. The effect of several risk factors on survival will be analyzed using Cox regression (or proportional hazards regression). A *P* value <0.05 will be considered statistically significant. All statistics will be processed using IBM SPSS version 22.0 software (IBM, Armonk, NY, USA) A fully specified statistical analysis plan will be written and approved at least before the database is unlocked.

## Discussion

We hypothesize that liver resection for HBV-related HCC without vascular occlusion is technically feasible and perhaps has long-term oncological benefits. To our knowledge, this is the first prospective RCT to date to compare the perioperative and long-term outcomes of liver resection for HBV-related HCC with versus without hepatic inflow occlusion (Pringle maneuver). Some points need to be discussed to clarify our study objective and the study’s clinical implications.

### Trial population

The high prevalence of HBV infection imposes a huge burden of cirrhosis and HCC in Asia. The World Health Organization reported that the western Pacific and Southeast Asian regions have the highest rates of HBV infection worldwide. The prevalence in these areas represents >75 % of the world’s HBV carriers and accounts for >60 % of the world’s liver cancer cases [30, 31]. It has been projected that, in the next decade, approximately 80 % of the world’s HCC cases will be in Asia [32]. RCTs on the Chinese population with HBV-related HCC will therefore have a greater clinical impact. We are in a unique position to enroll the necessary number of eligible patients.

The study is directed at only the patients with HBV-related HCC rather than all patients with HCC. The high prevalence of HBV infection in certain local areas has been taken into consideration. More importantly, restricting the underlying disease to HCC will help us to stratify the patients, reduce confounding variables, and improve the comparability of the groups. We surmise that hepatic inflow occlusion may induce a more severe hepatic IR injury and perioperative inflammatory response in patients with HBV-related HCC, owing to the underlying proinflammatory disease (hepatitis and/or cirrhosis). The results of the study may provide an answer to this concern and change the current practice for treatment of HBV-related HCC.

### Trial objective

Various techniques of vascular occlusion have been practiced to reduce blood loss during liver resection [33]. Hepatic inflow occlusion (the Pringle maneuver) is a long-standing method used because of its simplicity and proven efficacy [34]. A major concern with this maneuver is the IR injury to the remnant liver. As a common consequence of HBV infection, liver cirrhosis is detected in most cases of HBV-related HCC [10]. It is generally recognized that the cirrhotic liver is particularly sensitive to the IR injury [11, 12]. Given the technical advances in LDLT, the Pringle maneuver could be avoided in liver resection in experienced hands [17, 18]. All these considerations imply that liver resection without vascular occlusion could be a more preferred option for patients with HBV-related HCC. The clinical implications of the outcomes of the present study may change current clinical practice. Furthermore, the hemodynamic disturbance to the tumor-bearing liver by intermittent hepatic inflow occlusion remains an oncological concern [13, 14, 35, 36]. Long-term follow-up of patients with HCC in this study will provide clinical clues and perhaps fill current gaps in knowledge.

### Trial endpoints

Serum TBil has been used widely as an important parameter for determining PHLF. The ISGLS defines PHLF as an increased serum TBil on or after POD 5 or an increasing TBil (compared with values at the previous time point) if TBil is increased preoperatively (INR is an equivalent parameter in this criterion) [26]. The “50-50” criterion defines PHLF with PT >50 % (or INR >1.7) and TBil >50 μmol/L on POD 5 [37]. As a widely agreed prognostic factor, serum TBil on POD 5 is selected as the primary endpoint in the present study. In our pilot study, a 20 % difference in serum TBil on POD 5 was found between the occlusion and nonocclusion groups. Although the average TBil levels of both groups were within acceptable range (15.0 ± 5.3 μmol/L vs. 19.2 ± 6.9 μmol/L), the preliminary data indicated a potential difference in perioperative outcomes between the two groups, and its clinical significance might be reflected by other endpoints. These conclusions are based on analysis of retrospective data, which could have been influenced by some confounding variables. The future prospective RCT will provide more analyzable data.

The secondary endpoints include most perioperative and long-term parameters. We also added serum inflammatory factors, such as the IL-1, IL-2, IL-8, IL-10, TNF-α, and CRP. Hepatic IR injury results in a complex release of inflammatory mediators [38]; thus, the perioperative inflammatory response would reflect the impact of vascular occlusion and link it to the subsequent outcomes.

This is to date the first prospective RCT comparing liver resection for HBV-related HCC with versus without hepatic inflow occlusion. The results with respect to the perioperative and long-term outcomes will be analyzed and discussed upon the completion of data collection.

## Trial status

The study protocol was designed in June 2015 and registered with ClinicalTrials.gov (NCT02563158) in September 2015. Patient enrollment started in January 2016. Twenty six patients have been enrolled by September 2016.

## Additional files

Additional file 1: SPIRIT 2013 Checklist: recommended items to address in a clinical trial protocol and related documents. (DOC 123 kb)
Additional file 2: Figure S1. Schedule of enrollment, interventions, and assessments. (JPG 920 kb)

## Abbreviations

AFP: Alpha-fetoprotein
ALB: Albumin
ALT: Alanine aminotransferase
AST: Aspartate aminotransferase
CONSORT: Consolidated Standards of Reporting Trials
CRF: Case report form
CRP: C-reactive protein
CT: Computed tomography
HBV: Hepatitis B virus
HCC: Hepatocellular carcinoma
ICU: Intensive care unit
IL: Interleukin
INR: International normalized ratio
IR: Ischemia-reperfusion
ISGLS: International Study Group of Liver Surgery
LDLT: Living donor liver transplant
MRI: Magnetic resonance imaging
PCT: Procalcitonin
PHLF: Posthepatectomy liver failure
PLAGH: Chinese People’s Liberation Army General Hospital
POD: Postoperative day
PRBC: Packed red blood cell
PT: Prothrombin time
RCT: Randomized controlled trial
TBil: Total bilirubin
TNF-α: Tumor necrosis factor-α
