# Supplementary figures and images for: Perioperative and long-term outcomes of liver resection for hepatitis B virus-related hepatocellular carcinoma without versus with hepatic inflow occlusion: study protocol for a prospective randomized controlled trial
Source: Trials. 2016 Oct 11;17:492. doi: 10.1186/s13063-016-1621-9 (PMC5057253; doi:10.1186/s13063-016-1621-9)

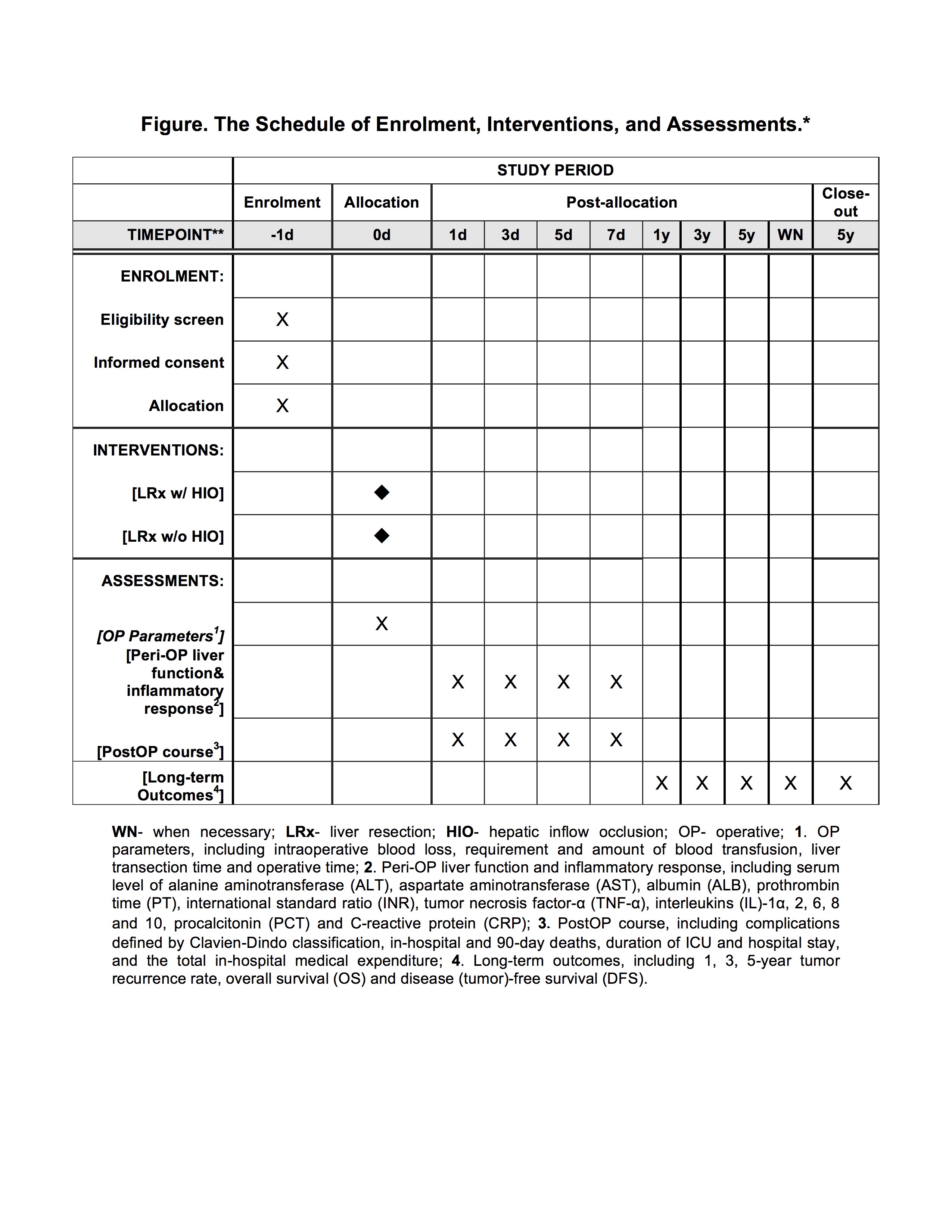

Supplement: Additional file 2: Figure S1. — Schedule of enrollment, interventions, and assessments. (JPG 920 kb) [file 13063_2016_1621_MOESM2_ESM.jpg]
